# Layered Monitoring of Ground Subsidence Based on Ultra-Weak FBG Sensing Technology: A Case Study in Gaoyang County, China

**DOI:** 10.3390/mi16121380

**Published:** 2025-12-04

**Authors:** Haigang Wang, Huili Gong, Jincai Zhang, Lin Zhu, Di Ning, Chaofan Zhou, Xingguang Yan

**Affiliations:** 1 College of Resource Environment and Tourism, Capital Normal University, Beijing 100048, China; w13521959046@163.com (H.W.);; 2 Hebei Cangzhou Groundwater and Land Subsidence National Observation and Research Station, Cangzhou 061000, China; 3 Hebei Geo-Environment Monitoring, Shijiazhuang 050021, China

**Keywords:** layered monitoring, ultra-weak fiber Bragg grating, ground subsidence, fiber-reinforced polymer, structural safety monitoring

## Abstract

The primary objective of layered settlement monitoring of deep soil is to obtain settlement data for both the soil and superstructure, enabling appropriate measures to be taken to ensure the structure’s safety and stability. Traditional deep soil monitoring technologies are either limited in the number of measurement points (e.g., fiber Bragg grating sensing technology) or exhibit low measurement accuracy (e.g., distributed fiber optic sensing technology). This study proposes a layered settlement monitoring technique for deep soil based on the ultra-weak fiber Bragg grating sensors. First, ultra-weak fiber Bragg grating strain sensors packaged by fiber-reinforced polymer (FRP) were developed, and experimental research on the sensors’ sensing and directional recognition characteristics was conducted. Subsequently, the sensors were deployed for ground subsidence monitoring in Gaoyang County, China, with investigations conducted on sensor installation techniques and long-term measurement data. Experimental and engineering test results demonstrate that the strain and temperature sensing coefficients of the sensors are 1.22 pm/με and 17.06 pm/°C, respectively. Sensors incorporating dual ultra-weak fiber Bragg grating arrays can simultaneously detect both vertical and lateral soil displacement. Long-term monitoring data effectively reflects subsidence changes in the Gaoyang region.

## 1. Introduction

With the rapid advancement of urbanization and industrialization, the demand for underground resources driven by human engineering activities has intensified. Among these, the large-scale and concentrated extraction of groundwater to meet production and daily water needs has become a primary cause of regional land subsidence. This geological process essentially occurs because after water is pumped from underground aquifers, the pore water pressure decreases and the effective stress increases, leading to the irreversible compaction and consolidation of the soil skeleton that supports the overlying strata. This widespread, cumulative subsidence deformation, while seemingly slow, poses a long-term and serious threat to urban buildings, transportation networks, flood control systems, and underground pipelines.

To accurately diagnose this “chronic urban ailment,” various methods are used for ground subsidence monitoring. The surface displacement monitoring technologies include InSAR (Interferometric Synthetic Aperture Radar), drone inspection, and automatic total station, which are primarily used for precise measurement of ground elevation subsidence or horizontal displacement [1,2,3,4,5,6]. However, surface displacement monitoring only reveals the total displacement at the surface, without indicating the depth or specific soil layer contributing to this result. Deep displacement monitoring primarily addresses the following questions: “Why is subsidence occurring? How is it developing? Where is the slip surface located?” Compared to surface displacement monitoring, deep monitoring can detect minute deformations deep underground before significant surface cracks or displacement becomes visible, enabling earlier warning. At present, local FBG sensing technology and distributed optical fiber technology have been used for deep soil layer displacement monitoring [7,8,9,10,11]. He et al. developed distributed optical fiber strain sensors packaged by FRP and successfully used for monitoring of foundation subsidence and deep displacement in slopes [12,13]. In these studies, FRP exhibits excellent corrosion resistance and mechanical properties. Encapsulating optical fibers or fiber gratings within FRP significantly enhances the sensor’s service performance. Specifically, the FBG sensors and distributed optical fiber sensors are vertically implanted into rock and soil layers at varying depths from the surface down to the primary aquifer. This enables a “three-dimensional analysis” of the subsidence process—we can not only determine the extent of ground subsidence but also clearly “see” at which depth and in which soil layer the subsidence is occurring, along with the contribution ratio of different soil layers to the total subsidence volume. However, local FBG sensing technology is limited by laser bandwidth, resulting in a finite number of measurement points, while distributed optical fiber Brillouin sensing technology exhibits relatively lower testing accuracy. Ultra-weak FBG technology is a novel monitoring technique developed in recent decades, which uses the fiber Rasta online preparation process to realize the grating array fibers with no melting point and high mechanical strength. Compared with the FBG and distributed sensing technologies, it has the advantages of both point sensing technology with high measuring accuracy and distributed sensing technology. At present, ultra-weak FBG sensors have been successfully applied in monitoring pipeline leaks, vehicle loads, slope stability, and bridge deflection deformation [14,15,16,17]. At the same time, we conducted a statistical analysis of the advantages and disadvantages of the aforementioned soil layer deformation monitoring or detection technologies using Table 1. Additionally, the FRP structure offers excellent corrosion resistance and superior mechanical properties. Encapsulating the ultra-weak FBG array within FRP effectively enhances the service performance of the ultra-weak FBG sensors.

To enable precise monitoring of ground vertical deformation, this paper investigates continuous deep displacement monitoring technology based on ultra-weak FBG sensors. First, two ultra-weak FBG arrays were embedded into FRP reinforcement to develop an FRP-packaged ultra-weak FBG strain sensor, and its sensing characteristics were studied through static tensile and temperature tests. Subsequently, in Gaoyang County, China, the sensor was deployed for long-term ground subsidence monitoring. Primarily through drilling and backfilling protection techniques, the sensor was embedded into bedrock at a depth of 350 m to conduct sustained observation studies on ground subsidence.

## 2. Sensing Principle of the Ultra-Weak FBG Sensor

Ultra-weak FBG is a kind of FBG with very weak reflectivity, which has the advantages of high-precision, high-sensitivity, long-distance, and distributed monitoring. The energy reflected by a single FBG is very low. When the FBG’s reflectivity is below −40 dB (0.1%), a single wavelength can support thousands of sensing points. The relationship between the correlated light intensity of a single FBG and the number of gratings and reflectance is as follows:

(1) $$\frac{I_{i}(\lambda )}{I_{0}(\lambda )}=R(\lambda )\left[1-R\left(\lambda \right)\right]2(i-1)$$

Here, $I_{i}(\lambda )$ is the light intensity after *i* FBG reflections; $I_{0}(\lambda )$ is the incident light intensity; and R(λ) is the reflectance [18].

When the fiber core is subjected to external axial strain or temperature changes, the center wavelength of the FBG undergoes drift. Research findings indicate that the center wavelength of the fiber Bragg grating exhibits a linear relationship with both temperature and strain as follows:

(2) $$\Delta \lambda_{B}=C_{\varepsilon }\Delta \varepsilon +C_{T}\Delta T$$

Here, $\Delta \lambda_{B}$ is the central wavelength variation induced by strain and temperature simultaneously; $C_{\varepsilon }$ and $C_{T}$ are the strain and temperature sensing coefficients, respectively; and Δε and ΔT are the strain and temperature variations.

Given that the FBG’s center wavelength is modulated by both ambient temperature and strain, temperature compensation for strain measurements is required in practical applications. The temperature compensation method involves placing a temperature sensor near the strain sensor. The specific compensation process is described by Equations (3) and (4):

(3) $$\Delta \lambda_{T}=C_{TT}\Delta T$$

Equation (3) is the sensing expression for the temperature sensor. Here, $\Delta \lambda_{T}$ is the central wavelength variation induced by temperature and $C_{TT}$ is the temperature variation. By combining Equations (2) and (3), the influence of temperature on strain measurement can be eliminated, as shown in Equation (4) [19,20]:

(4) $$\Delta \varepsilon =\left(\Delta \lambda_{B}-C_{T}\frac{\Delta \lambda_{T}}{C_{TT}}\right)/C_{\varepsilon }$$

## 3. Development and Performance Testing Research of Ultra-Weak FBG Sensor Packaged by FRP

**Figure 1:**
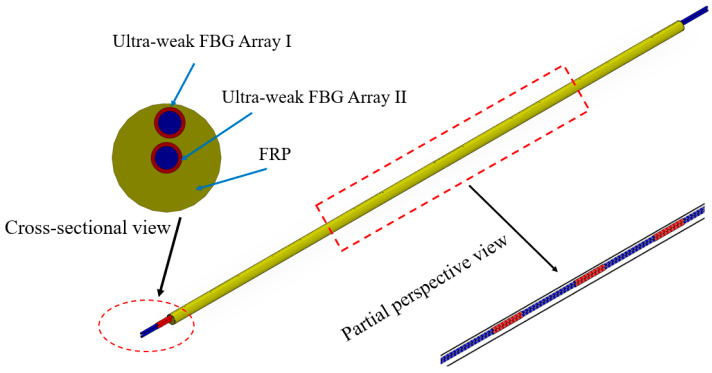
The schematic diagram of the sensor‘s structure.

Given the slender diameter and poor shear resistance of bare fiber gratings, they are unsuitable for direct use as sensors in engineering testing, particularly when deeply embedded in rock and soil layers. Additionally, considering sensor durability, we employed fiber-reinforced resin encapsulation for the ultra-weak FBGs to enhance tensile strength, shear resistance, and service life. Figure 1 shows the schematic diagram of the sensor‘s structure. The sensor is named FRP-UWFBGN-M sensor (manufactured by Dalian Boruixin Technology Park Co., Ltd. in Dalian, China), which includes one ultra-weak FBG array I, one ultra-weak FBG array II, and FRP. Here, the jumper head of the sensor is not shown, and the uppercase letters N and M in the sensor name denote the number of ultra-weak FBGs and the spacing between weak fiber grating measurement points within a single ultra-weak FBG array, respectively. Ultra-weak FBG array I and ultra-weak FBG array II are embedded parallel to each other within the FRP reinforcement during the FRP pultrusion process. Ultra-weak FBG array I is positioned at the edge of the FRP reinforcement, primarily serving to identify the direction of lateral deformation, and ultra-weak FBG array II is positioned along the central axis of the FRP reinforcement, primarily serving to measure structural deformation. The spacing and number of the ultra-weak FBGs are determined by monitoring requirements. The FRP-UWFBG sensor is manufactured using the FRP pultrusion process and has a diameter of 5.0 mm. The FRP may be GFRP (glass fiber reinforced polymer), CFRP (carbon fiber reinforced polymer), or BFRP (basalt fiber reinforced polymer). The FRP used in this study is GFRP, and its elastic modulus, shear strength, and ultimate tensile strain are 45 GPa, 740 N, and 1.6%, respectively [21].

**Figure 2:**
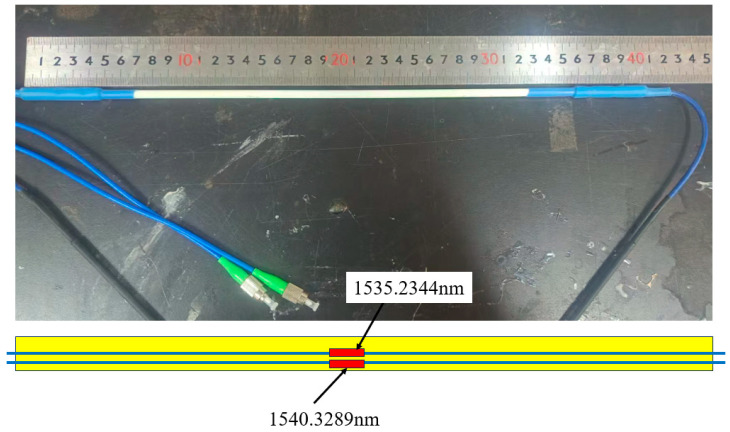
The photograph of the FRP-UWFBG1-0 sensor.

Figure 2 shows the photograph of the developed sensor named FRP-UWFBG1-0. The length and the diameter of the sensor are 400 mm and 5 mm, respectively. Both ultra-weak FBG array I and ultra-weak FBG array II are included in one ultra-weak FBG.

**Figure 3:**
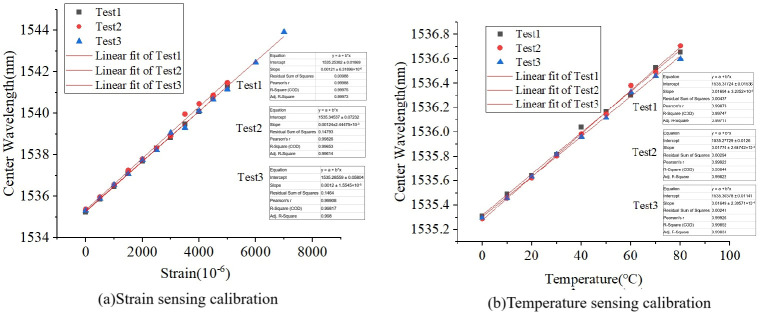
The strain and temperature calibration results.

Figure 3 shows the strain and temperature calibration results for FRP-UWFBG1-0. The strain test is an axial tensile test, so we only used the ultra-weak FBG on the central axis for strain and temperature calibration. The strain and temperature calibration experiments were each conducted three times, with a strain increment step size of 500 με and a temperature increment step size of 10 °C. Table 2 lists the strain sensing coefficients and temperature sensing coefficients for the three tests, respectively. It can be seen that the average strain and temperature sensing coefficients are 1.22 pm/με and 17.06 pm/°C, respectively, and all linear correlations reached values above 0.999.

**Figure 4:**
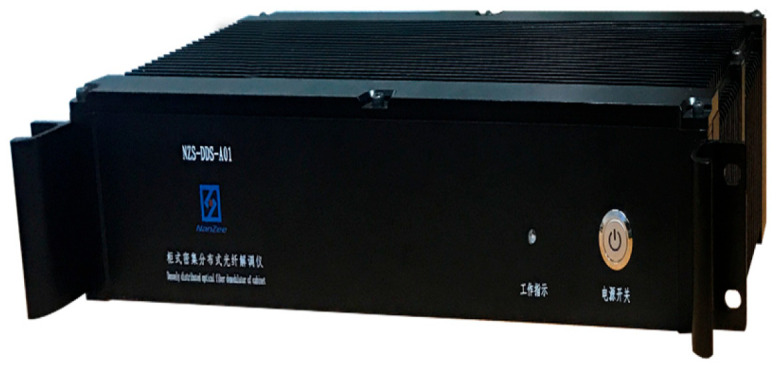
Data acquisition device (Model NZS-DOS-A01).

Figure 4 shows the data acquisition device, model NZS-DOS-A01, with a spatial resolution of 500 mm, wavelength measurement range of 1525–1565 nm, and accuracy of 1.5 pm.

**Figure 5:**
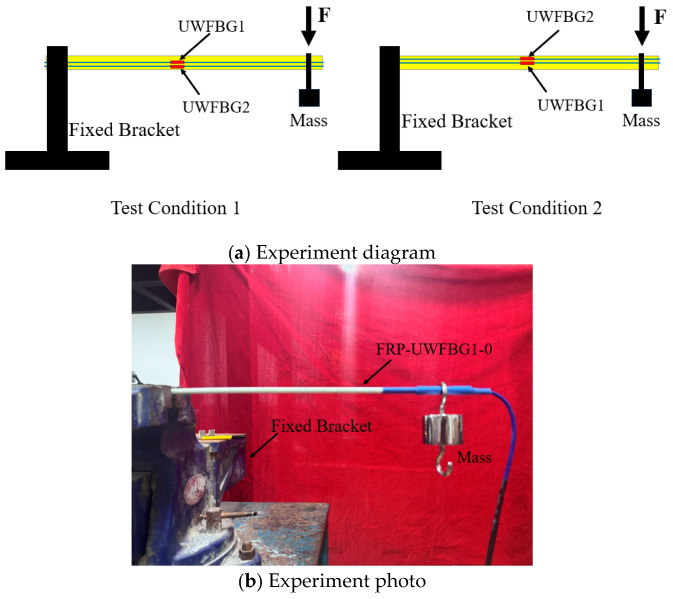
Experimental setup.

Figure 5 shows the schematic diagram and photograph of the sensor direction recognition test. In the figure, one end of the sensor is fixed to the bracket, while the other end suspends a mass block. In the sensor, the ultra-weak FBG on the sensor’s central axis is designated as UWFBG1, and the direction-sensing ultra-weak FBG is designated as UWFBG2. We established two test conditions as shown in Figure 5a: Condition 1: the direction of force is closer to the UWFBG1 side; Condition 2: the direction of force is closer to the UWFBG2 side. Each operating condition includes two load levels: the first load level has a mass of 200 g, and the second load level has a mass of 700 g. In fact, the first and second test conditions represent two distinct scenarios where the force applied to the sensors is directed oppositely. Theoretically, under the first test condition, the strain measured by UWFBG1 is positive and increases with increasing load; the strain measured by UWFBG2 is negative and decreases with increasing load. Under the second test condition, due to the change in the direction of force application, both the strain measured by UWFBG2 and UWFBG1 are positive and increase with increasing load.

Table 3 lists the variation in center wavelength of the two ultra-weak FBGs in the sensor. It can be seen that the center wavelength of UWFBG1 increases with increasing load and the center wavelength of UWFBG2 decreases with increasing load under the first condition. Under the second condition, the center wavelength of UWFBG2 increases with increasing load and the center wavelength of UWFBG1 decreases with increasing load. The reason the measurements measured by UWFBG1 do not match the theoretical conditions is that UWFBG1 is not positioned on the central axis. Based on the measured data, it can be determined that UWFBG1 and UWFBG2 are located on opposite sides of the central axis.

**Table 3:** Sensor direction recognition test results.

	Test Condition 1		Test Condition 2	
Load	UWFBG1/nm	UWFBG2/nm	UWFBG1/nm	UWFBG2/nm
0	1540.329	1535.234	1540.332	1535.234
200 g	1540.391	1535.131	1540.253	1535.28
700 g	1540.526	1534.913	1540.095	1535.391

Due to the small diameter of the sensor, it is impossible to strictly ensure that UWFBG1 is positioned on the sensor’s central axis during manufacturing. Therefore, when determining the direction of lateral force applied to the sensor, the following three scenarios may occur. (1) When UWFBG1 and UWFBG2 are positioned on opposite sides of the sensor’s central axis, if the measured value of UWFBG1 increases with increasing load while the measured value of UWFBG2 decreases with increasing load, this indicates that the lateral force direction acts on the UWFBG1 side. (2) When both UWFBG1 and UWFBG2 are positioned on the same side of the sensor’s central axis, the sensor closer to the lateral load will exhibit a higher measured value than the other sensor. (3) When UWFBG1 is on the central axis and UWFBG2 is on one side of the central axis, the measurement value of UWFBG1 consistently increases with increasing load. If the lateral load is on the UWFBG2 side, the measurement value of UWFBG2 increases with increasing load. If the lateral load is on the opposite side of UWFBG2, its measured value decreases as the load increases.

However, in either case, we can determine the direction of the lateral load by analyzing how the UWFBG1 and UWFBG2 measurements change with load.

## 4. Ground Layered Settlement Monitoring: A Case Study in Gaoyang, China

The subsidence zone in Gaoyang County, China, located in the southern part of the Xiong’an New Area, is the region with the highest ground subsidence rate in the plains of the Hebei Province. The subsidence disaster has severely impacted the production and daily lives of nearby residents, with some houses exhibiting subsidence-induced cracks. To monitor the long-term development of ground subsidence in this area, track the vertical compression distribution of strata, and investigate the mechanisms causing regional subsidence, the ultra-weak FBG sensing technology was employed for layered subsidence monitoring of the ground in this region. This monitoring area features one borehole with a depth of 350 m. Through analysis of the drilled soil samples, the distribution of the soil layer’s physical properties was obtained as listed in Table 4, which shows that the physical properties of soil layers at different depths in this area exhibit good consistency. Considering monitoring effectiveness and cost, the spacing between weak fiber optic grating monitoring points is set to 5 m. So, the sensor is named FRP-UWFBG58-5, which contains a total of 58 measurement points or ultra-weak FBGs.

**Figure 6:**
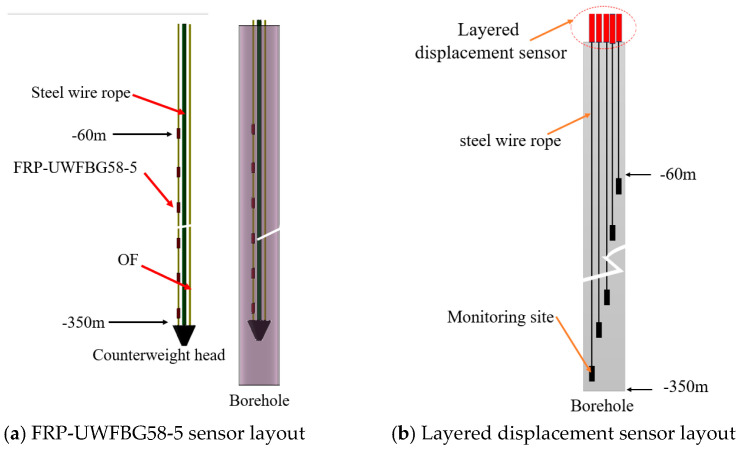
FRP-UWFBG58-5 sensor and layered displacement sensor deployment diagrams.

The FRP-UWFBG58-5 sensor is deployed using a suspended weight system at the borehole tip as shown in Figure 6a. Layered displacement sensors are also installed near this borehole, which enable uniform measurement across all strata segments, while the ultra-weak FRP-UWFBG58-5 sensor achieves full-process, distributed, and precise deformation capture within the borehole. Based on the analysis of soil samples collected from the drilled boreholes, the lithology in this area predominantly consists of silt, silty clay, clay, sandy clay, medium to fine sand of varying grain sizes, and silt sand. The stratigraphic structure is primarily characterized by alternating layers of sandy soil and cohesive soil. After sensor installation is complete, backfill and seal the boreholes using the aforementioned sampling materials. Figure 6b shows a schematic diagram of the layered displacement sensor deployment. The layered displacement sensor is actually composed of multiple cable-type displacement sensors. The first monitoring points of the FRP-UWFBG58-5 sensor and layered displacement sensor were located 60 m below the surface.

**Figure 7:**
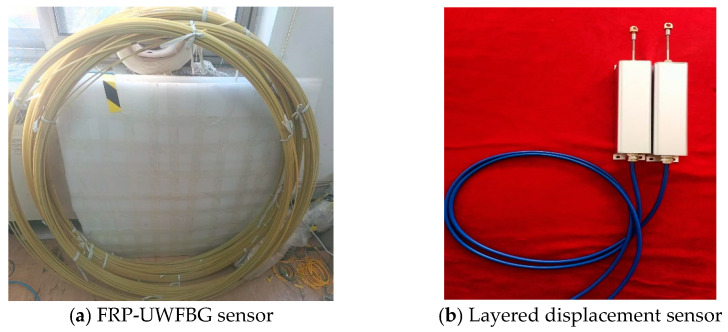
The photos of the FRP-UWFBG58-5 sensor and layered displacement sensor.

**Figure 8:**
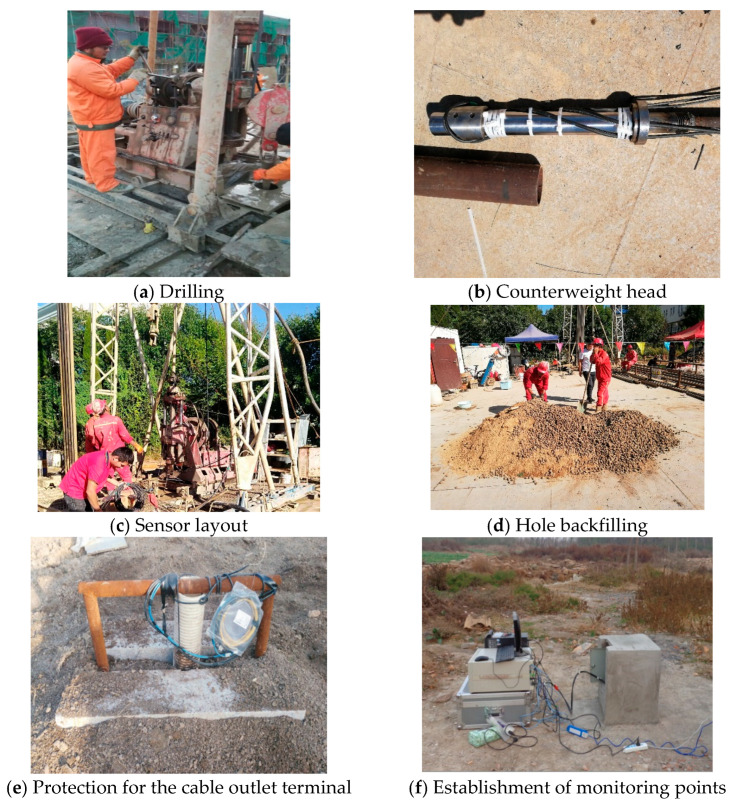
Sensor layout process.

Figure 7 shows the physical images of the layered displacement sensor and the FRP-UWFBG58-5 sensor. The accuracy and measurement range of the layered displacement sensor are ±0.5 mm and 50 mm, respectively, and the accuracy and measurement range of the FRP-UWFBG58-5 sensor are ±0.1 mm and 30 mm, respectively. Figure 8 shows photographs of the sensor field deployment. The installation process comprises six steps: drilling, counterweight head installation, sensor deployment, borehole backfilling, sensor cable end protection, and monitoring station establishment. The mass of the counterweight head is 1000 g. The FRP-UWFBG58-5 sensor, optical fiber (OF), and steel wire rope are secured to the counterweight head, ensuring the sensor remains in a straight line at all times. Based on long-term underground temperature observation data, temperatures at depths ranging from 46 to 500 m below the surface remain largely unaffected by seasonal temperature fluctuations and are essentially constant. Therefore, this monitoring plan does not include the installation of temperature sensors for environmental temperature compensation. Furthermore, when the length of the FRP-UWFBG58-5 sensor reaches 350 m, multiple UWFBG sensors detect the same temperature field within a certain range, and since at least one UWFBG sensor is not affected by soil deformation, this sensor can serve as a temperature sensor to compensate for the temperature of other sensors.

**Figure 9:**
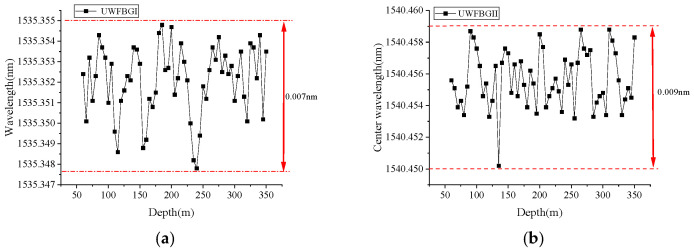
The center wavelength distribution of the two ultra-weak FBG arrays in the FRP-UWFBG58-5 sensor. (**a**) The center wavelength distribution of UWFBGI at the initial state, (**b**) the center wavelength distribution of UWFBGII at the initial state.

**Figure 10:**
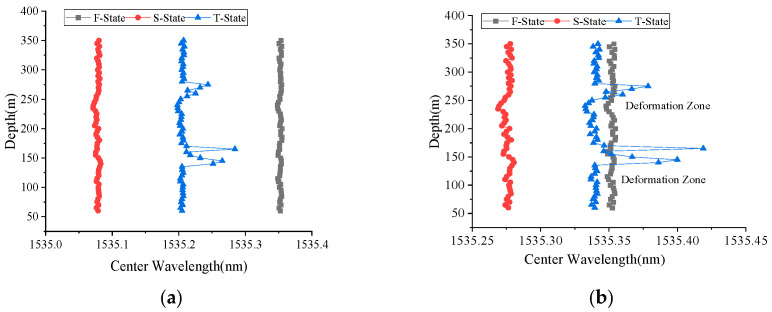
Center wavelength distribution of UWFBGI in the FRP-UWFBG58-5 sensor. (**a**) Center wavelength distribution without temperature compensation, (**b**) center wavelength distribution with temperature compensation.

**Figure 11:**
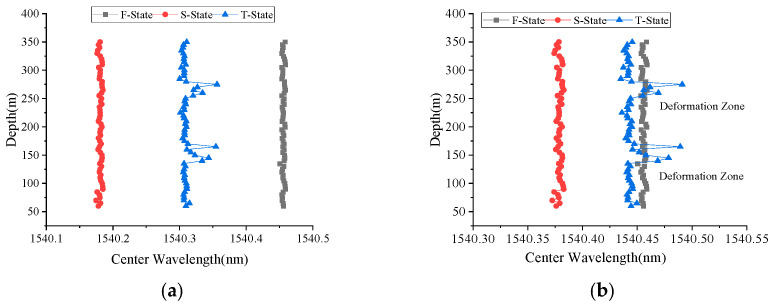
Center wavelength distribution of UWFBGII in the FRP-UWFBG58-5 sensor. (**a**) Center wavelength distribution without temperature compensation, (**b**) center wavelength distribution with temperature compensation.

In the FRP-UWFBG58-5 sensor, there are two ultra-weak FBG arrays: one is for displacement measurement, named UWFBGI, and the other is for direction identification, named UWFBGII. Figure 9 shows the center wavelength distribution of the two ultra-weak FBG arrays in the FRP-UWFBG58-5 sensor; it can be seen that the center wavelengths of the fiber gratings within each weak-light fiber grating array are essentially identical, with the UWFBGI array having a center wavelength range of [1535.3476 nm, 1535.3551 nm] and the UWFBGII array having a center wavelength range of [1540.4503 nm, 1545.4592 nm]. Figure 10 and Figure 11 show the center wavelength distribution of the ultra-weak FBGs in the FRP-UWFBG58-5 sensor before and after temperature compensation at three different states. The first state (named as F-State) is when the sensor was outdoors in a free state and the outdoor ambient temperature was 32.3 °C; the second state (named as S-State) is when the FRP-UWFBG58-5 sensor was installed into the borehole, and the borehole was not backfilled, and the borehole temperature was 20.7 °C; and the third state (named as T-State) is at twenty-four hours after backfilling the borehole and the temperature inside the hole was 24.4 °C. Here, traditional resistance temperature detectors such as the PT100 are used for ambient and borehole temperature measurements.

**Figure 12:**
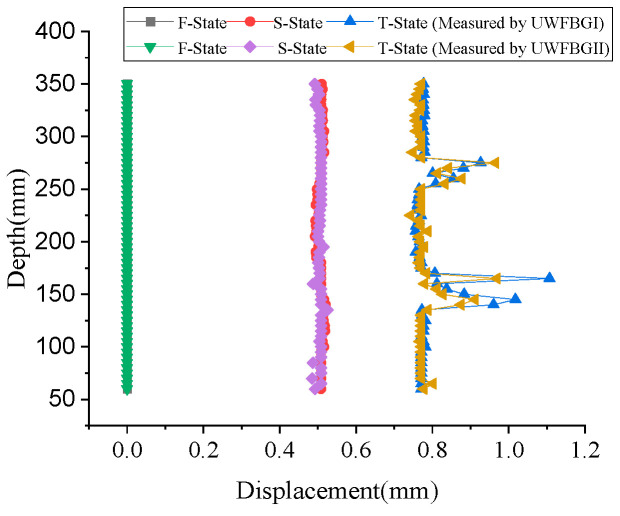
The displacement distribution measured by the FRP-UWFBG58-5 sensor.

Figure 12 shows the displacement distribution measured by the FRP-UWFBG58-5 sensor. The displacement ( $D_{i}$) is calculated from strain ( $\varepsilon_{i}$), as shown in Equation (5).

(5) $$D_{i}=\varepsilon_{i}\times 5000$$

Here, $D_{i}$ and $\varepsilon_{i}$ are the displacement and strain of ith m. Since the accuracy of the data acquisition device is 1.5 pm, this translates to a strain of 1.25 με. Therefore, the vertical displacement accuracy within a 5 m length range is 0.006 mm.

It can be seen that the sensors experienced lateral load between soil layers 250–300 m and 130–160 m after backfilling the borehole. At the 130 m to 160 m position, since the displacement value measured by UWFBGI is greater than that measured by UWFBGII, it can be determined that the lateral load is closer to the side of UWFBGI. Since at the 250 m to 300 m position, the displacement value measured by UWFBGII is slightly greater than that measured by UWFBGI, it also can be determined that the lateral load is closer to the side of UWFBGII.

**Figure 13:**
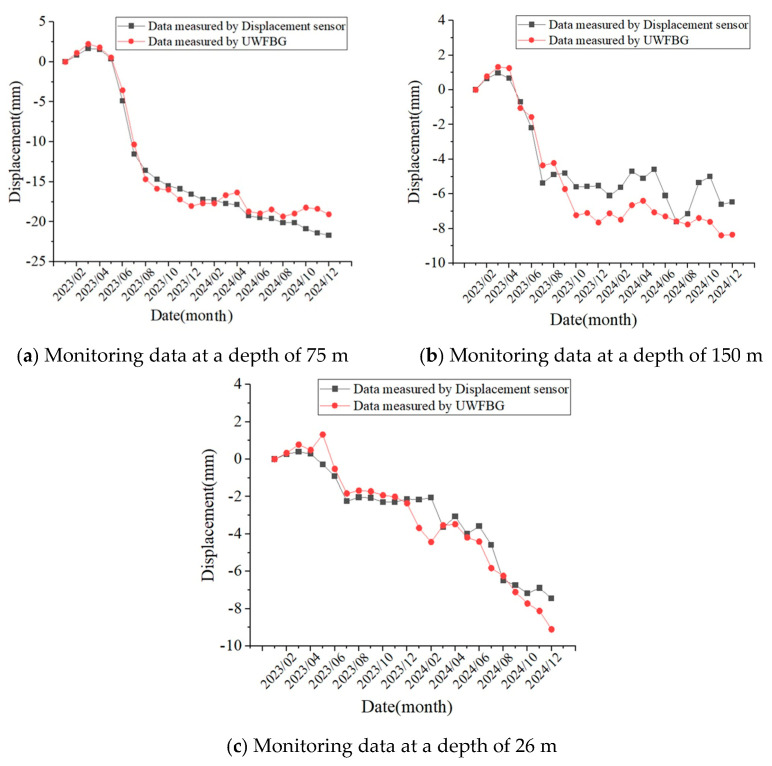
The displacement comparison curves between the layered displacement sensor and the FRP-UWFBG58-5 sensor.

**Figure 14:**
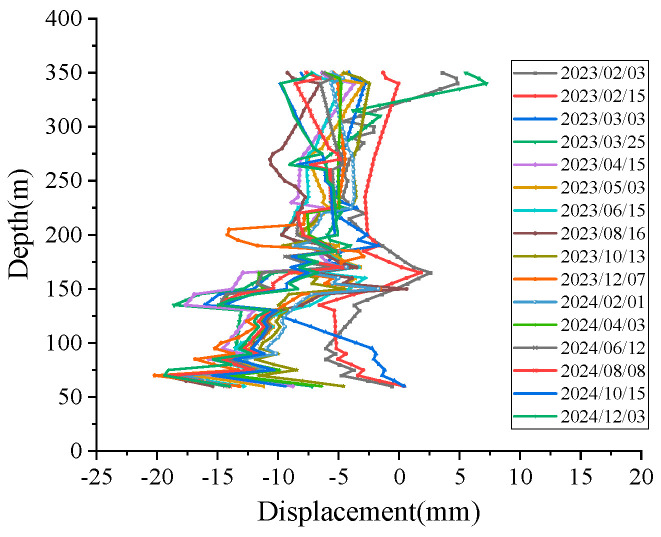
The distribution of deep soil displacement from February 2023 to December 2024.

One month after backfilling the borehole, the backfill material within the hole became substantially compacted under its own weight and lateral loading from the surrounding soil, achieving tight coupling with the in situ soil. We initiated long-term monitoring of soil settlement using data from the first test conducted on 3 February 2023 as the baseline value. In subsequent testing, since only the displacement sensors at the 75 m, 150 m, and 265 m points operated normally, we conducted comparative analysis solely on the displacement measurements at these three locations. Figure 13 shows the displacement comparison curves. Monitoring data from the FRP-UWFBG58-5 sensor and layered displacement sensor both indicate that from January to March in 2023, the strata were in a rebound state with settlement rates tending to decrease. After May in 2023, the strata entered a compression state, with settlement rates gradually increasing. The trends observed in the monitoring data from the FRP-UWFBG58-5 sensor and layered displacement sensors were largely consistent. The final settlement values measured by the layered displacement sensor are −21.67 mm, −6.48 mm, and −6.45 mm at the 75 m, 150 m, and 265 m locations, and those measured by the FRP-UWFBG58-5 sensor are −19.07 mm, −8.35 mm, and −9.10 mm as listed at the 75 m, 150 m, and 265 m locations in Table 5. One of the primary reasons for the discrepancy between monitoring data from the ultra-weak FBGs and layered displacement sensor stems from lateral deformation of the ultra-weak fiber optic grating sensors caused by lateral compression from backfill material. Figure 14 shows the distribution of deep soil displacement from February 2023 to December 2024, with sampling points spaced at 5 m intervals measured by the FRP-UWFBG58-5 sensor. Overall, the test results above indicate that both the layered displacement sensor and the FRP-UWFBG58-5 sensor demonstrate high accuracy in fitting stratum deformation patterns, both capable of intuitively and accurately reflecting soil deformation characteristics. The unique advantage of the ultra-weak FBG array lies in its ability to characterize stratum changes across the entire pore space and cross-section, enabling analysis of deformation patterns at any stratum level.

## 5. Conclusions

In this paper, we proposed layered subsidence monitoring for the ground based on ultra-weak FBG sensing technology. To enhance the sensor’s survivability and enable lateral deformation direction recognition, two ultra-weak FBG arrays were simultaneously encapsulated within FRP material. The sensor’s sensing performance was experimentally investigated. Ultimately, the developed sensor was successfully deployed for long-term ground subsidence monitoring in Gaoyang, China, where the monitoring data demonstrated excellent consistency with existing layered displacement sensor measurements. Perception performance tests and field data demonstrate that the developed sensor exhibits excellent engineering characteristics, making it suitable for monitoring vertical layered ground subsidence. Key achievements include the following:

(1) The FRP-UWFBGN-M sensor exhibits excellent sensing characteristics, with strain sensitivity coefficients and temperature sensitivity coefficients of 1.22 pm/με and 17.06 pm/℃, respectively, and the linear correlation coefficient reached above 0.999. By analyzing the strain difference between two ultra-weak FBGs at the same position within the developed sensor, the lateral deformation direction of the sensor can be identified.

(2) The FRP-UWFBG58-5 sensor was successfully deployed into deep soil layers to conduct layered settlement monitoring in Gaoyang County, China, and the monitoring range was from −60 m to −350 m. Compared to existing layered displacement sensors, the monitoring data measured by the FRP-UWFBG58-5 sensor effectively reflects settlement deformation across various zones.

Current experimental results primarily focus on monitoring and analyzing soil settlement trends. In subsequent research or engineering monitoring, pore water pressure sensors can be employed to obtain pore water pressure data at different locations within the soil. Based on settlement measurement data and pore water pressure sensor readings, detailed monitoring and assessment of soil settlement can be conducted.

## Figures and Tables

**Table 1:** The comparison of soil deformation monitoring technologies.

Monitoring Technology	Measurement Accuracy	Measurement Distance	Measurement Type	Measurement Method
InSAR	high	Large-scale	Surface Deformation Monitoring	Surface Monitoring
Automatic total station	high	Small	Surface Deformation Monitoring	Point monitoring
FBG strain tube	high	Long-distance	Lateral Deformation Monitoring	Quasi-distributed measurement
BOTDR/BOTDA	low	Long-distance	Settlement Monitoring	Continuous measurement
OFDR	high	Short distance (less than 200 m)	Settlement Monitoring	Continuous measurement
Ultra-weak FBG	high	Long-distance	Settlement Monitoring	Continuous measurement

**Table 2:** The strain and temperature sensing coefficients of the sensor.

	Test 1	Test 2	Test 3	Average
Strain sensing coefficients (pm/με)	1.21	1.24	1.20	1.22
Temperature sensing coefficients (pm/°C)	16.94	17.74	16.49	17.06

**Table 4:** The physical properties of soil layers.

Depth (m)	Physical Properties					
	Moisture Content (%)	Pore Ratio (e)	Dry Density (g/cm^3^)	Compression Coefficient (Mpa^−^^1^)	Plasticity Index (I_p_)	Proportion (kg/m^3^)
0–60	24.24%	0.69	1.61	0.21	14.39	2.73
60–170	19.82%	0.64	1.74	0.11	18.21	2.74
160–250	17.23%	0.58	1.78	0.09	21.65	2.75
250–350	17.08%	0.52	1.82	0.10	21.68	2.75
Average	18.61%	0.58	1.74	0.11	19.76	2.74

**Table 5:** The final settlement values at the 75 m, 150 m, and 265 m locations in two years (unit/mm).

Position	75 m	150 m	265 m
Layered displacement sensor	−21.67	−6.48	−6.45
FRP-UWFBG58-5	−19.07	−8.35	−9.1
Absolute error	−2.6	1.87	2.65

## Data Availability

The data presented in this study are available on request from the corresponding author.

## References

[1] Gogoi A., Kothyari G.C., Patidar A.K. (2025). Ground subsidence monitoring in India using InSAR: A review of current status and future prospects. Geosyst. Geoenviron..

[2] Zhang Z., Lin Q., Yan H., Zhu X., Zhang X., Yang S., Huang C., Shi Y., Hu J., Li X. (2025). Ground subsidence monitoring and prediction based on InSAR and CNN-BiGRU-Attention: A case study of Chengdu. Eng. Geol..

[3] Vishweshwaran M., Sujatha E.R. (2025). A review on applications of drones in geotechnical engineering. Indian Geotech. J..

[4] Akbar M., Pamuttu D.L., Budianto E., Rachmat R., Mardiyadi Z. (2025). Road Damage Identification Using a Combination of UAV Quadcopter Technology and Subgrade Investigation. Int. J. Saf. Secur. Eng..

[5] Tao C. (2024). Application of Automatic Monitoring of Static Leveling for Roadbed Settlement in Station Reconstruction Construction. Railw. Investig. Surv..

[6] He Y., Li Y., Xu L. (2024). An integrated multisource and multiscale monitoring technique for assessing the health status of high-speed railway subgrade. Remote Sens..

[7] Xu D., Jiang L., Qin Y., Shen H., Ji B. (2024). High-precision FBG-based sensor for soil settlement monitoring: A comparative study with magnetic settlement gauges and PIV technique. Sens. Actuators A Phys..

[8] Li C., Wei L., Xu Q., Yang L., Li J., Wan X. (2024). Structural Detection and Stability Monitoring of Deep Strata on a Slope Using High-Density Resistivity Method and FBG Strain Sensors. Appl. Sci..

[9] Sun D., Mao J., Liu M., Liu H., Zhang S., Li B., Jiang X., Ma J. (2025). A fiber Bragg grating (FBG)-strain sensing tube for deep displacement measurement. Opt. Laser Technol..

[10] Xue Y., Dai W., Wang X., Li C., He J. (2024). Development of OFDR-based inclination sensor for deformation measurement of foundation pile. Measurement.

[11] Wang J., Wang D., Zhu H.-H., Guo Z., Yan D., Tan D.-Y. (2024). Subsurface multi-physical monitoring of urban development zone using a fiber optic nerve system. J. Rock Mech. Geotech. Eng..

[12] Dai W., Xue Y., Wang X., Liu W., He J. (2025). Stability monitoring of deep soil in slope based on local strain and continuous vibration information analysis. Opt. Fiber Technol..

[13] He J., Xue Y., Xu J., Zhang D., Zhang S. (2020). Whole-process monitoring of sinkhole collapse based on distributed optical fiber strain-vibration joint system and its case study in railway subgrade. Opt. Fiber Technol..

[14] Chen J., Li Q., Zhang S., Lin C., Wei S. (2024). Convolutional autoencoder-based damage detection for urban railway tracks using an ultra-weak FBG array monitoring system. IEEE Sens. J..

[15] Nan Q., Yin S., Kang Y., Zeng J., Li S., Yue L., Yang Y. (2025). A Study on the Monitoring and Response Mechanism of Highway Subgrade Structures Based on Ultra-Weak FBG Sensing Array. Appl. Sci..

[16] Liu C., Wang Y., Tang K., Tang J., Cheng C., Yang M. (2025). Distributed multi-parameter sensing using composite optical fibers of hybrid ultra-weak fiber Bragg gratings. Opt. Laser Technol..

[17] Wang J., Yang Z., Wang S., Gao L., Song J. (2025). Large-capacity temperature points monitoring of lithium-ion battery pack via ultra-weak fiber Bragg grating array. Measurement.

[18] Huang J., He Y., Wang X., Gao J., Luo Z. (2025). Research on distributed strain sensing optical cable based on UW-FBG. Transducer Microsyst. Technol..

[19] Liu B., Li C., Zhang S., He J. (2020). Strain monitoring of laminate glass trestle by fiber Bragg grating sensors. Optik.

[20] He J., Qin T., Zhang Z., Liu R., Bao Y. (2025). Safety monitoring method for pipeline crossing the mining area based on vibration-strain fusion analysis. Micromachines.

[21] He J. (2010). Full-Scale Distributed Monitoring Technology of Optical Fiber Brillouin and Applications in Civil Engineering. Ph.D. Thesis.

